# Comparison of myxobacterial diversity and evaluation of isolation success in two niches: Kiritimati Island and German compost

**DOI:** 10.1002/mbo3.325

**Published:** 2015-12-15

**Authors:** Kathrin I. Mohr, Marc Stechling, Joachim Wink, Elke Wilharm, Marc Stadler

**Affiliations:** ^1^ Microbial DrugsHelmholtz Centre for Infection ResearchInhoffenstrasse 7BraunschweigD‐38124Germany; ^2^ Microbial Strain CollectionHelmholtz Centre for Infection ResearchInhoffenstrasse 7BraunschweigD‐38124Germany; ^3^ Department of Supply EngineeringOstfaliaSalzdahlumer Straße 46/48WolfenbüttelD‐38302Germany

**Keywords:** Biodiversity, environmental microbiology, myxococcales, secondary metabolites, taxonomy.

## Abstract

Myxobacteria harbor an enormous potential for new bioactive secondary metabolites and therefore the isolation of in particular new groups is of great interest. The diversity of myxobacteria present in two ecological habitats, namely sand from Kiritimati Island and German compost, was evaluated by both cultivation‐based and cultivation‐independent methods. Phylogenetic analyses of the strains in comparison with 16S rRNA gene sequences from cultured and uncultured material in GenBank revealed a great potential of undescribed myxobacteria in both sampling sites. Several OTUs (operational taxonomic units) represent unknown taxa and were detected by clone bank analyses, but not by cultivation. Clone bank analyses indicated that the myxobacterial community is predominantly indigenous. The 16S rDNA libraries from the two samples were generated from total community DNA with myxobacterial specific forward and universal reverse primer sets. The clones were partially sequenced. Cultivation was successful for exclusively bacteriolytic, but not for cellulolytic myxobacteria and revealed 42 strains from the genera *Corallococcus*,* Myxococcus*, and *Polyangium*. The genera of Myxococcaceae family were represented by both approaches. But, even in this well studied family, as well as in the suborders Sorangiineae and Nannocystineae, a considerable number of clones were assigned to, if any, uncultivated organisms. Our study shows an overrepresentation of the genera *Myxococcus* spp. and *Corallococcus* spp. with standard cultivation methods. However, high deficits are demonstrated in the cultivation success of the myxobacterial diversity detected by exclusively cultivation‐independent approaches. Especially, clades which are exclusively represented by clones are of high interest with regard to the cultivation of new bioactive secondary metabolite producers.

## Introduction

Myxobacteria are gram‐negative delta‐proteobacteria, and for several reasons are one of the most interesting groups of microorganisms: They show unique cooperative social behavior, based on a communication system of cell‐to‐cell interaction. Myxobacteria move by gliding on solid surfaces and use exoenzymes to lyse different biological macromolecules as well as whole organisms, such as bacteria or yeasts (Dawid 2000). Members of the order Myxococcales are masters of survival because in times of nutritional deficiency, they start to form species‐specific fruiting bodies, in which vegetative cells transform to dry‐resistant myxospores. Even after several years of storage, those spores are able to germ on suitable substrates. Another exceptional feature of myxobacteria is their enormous potential to produce bioactive secondary metabolites (Weissman and Müller 2010). To date, more than 100 new metabolites were isolated and described from this fascinating group. For example, epothilones are a class of microtubule‐targeting drugs (Gerth et al. 1996) that include ixabepilone, a semisynthetic epothilone derivative used for the treatment of breast cancer (Reichenbach and Höfle 2008).

The global distribution of myxobacteria is well reviewed by Dawid (2000) who evaluated data given in the literature on the basis of 1398 soil samples from 64 countries of all continents. Kiritimati (formerly Christmas) Island is the world's largest coral atoll within the Central Pacific and part of the Northern Line Islands of Republic of Kiribati (Woodroffe and McLean 1998). Kiritimati has 390 km^2^ of land, 324 km^2^ of lagoon, and 5500 inhabitants (Dinsdale et al. 2008). The sandy soil sample was taken near to the hypersaline Lake 21 (Schneider et al. 2013). Different studies dealt with the characterization of microbial communities and mats from hypersaline lakes of Kiritimati (Dinsdale et al. 2008; Schneider et al. 2013; Bühring et al. 2009) but, however, to the best of our knowledge, myxobacterial communities have never been detected, described, or investigated before in sand of this extraordinarily interesting location.

Composting involves the biological decomposition of organic matter under aerobic conditions to produce humus‐like product (Vaz‐Moreira et al. 2008). Bacterial communities in compost have been well studied for many years and revealed that this habitat harbors one of the largest reservoirs of a yet unexplored microbial diversity (Partanen et al. 2010). Moreover, numerous studies proved that myxobacteria are naturally present in compost (Singh 1947; Tanahashi et al. 2005; Sanford et al. 2002). Sanford et al. (2002) isolated and described the first and up to date only facultative anaerobic myxobacterium *Anaeromyxobacter dehalogenans* from, among other samples, Michigan yard compost. Tanahashi and co‐workers assumed that myxobacteria, together with other bacteria, are responsible for the decomposition process of rice straw compost (Tanahashi et al. 2005).

Compost is also a promising habitat for natural product producers: Cytostatic tubulysins (Sasse et al. 2000), as well as indothiazinones (Jansen et al. 2014) are produced by myxobacteria isolated from different German compost heaps. However, most bacteria remain uncultured and uncharacterized because appropriate culture conditions remain to be found (Vaz‐Moreira et al. 2008). These uncultured bacteria make up approximately 99% of all species in external environments, and are an untapped source of new antibiotics (Lewis 2013). Cultivation‐based techniques have allowed merely a glimpse of the microbial diversity because only an estimated 1% of the naturally occurring bacteria has been isolated and characterized so far (Muyzer 1999). Therefore, in addition to cultivation, we used a culture‐independent approach, based on PCR amplification and sequencing of 16S rRNA genes to assess and compare the diversity of myxobacteria detectable with these methods in two totally different samples.

In the past, it turns out that in particular, species of new phylogenetic lineages are reliable sources for new compounds (Jansen et al. 2014; Plaza et al. 2012; Steinmetz et al. 2012). Although descriptions of unknown myxobacteria, detected with standard cultivation on water agar with *E. coli* bait (Mohr et al. 2012; Sood et al. 2014; Garcia et al. 2014) or enhanced standard methods (soil samples suspended in sterilized water, serially diluted and plated onto BBPC‐VNC; Yamamoto et al. 2014) are regularly published, the optimization of particular cultivation experiments to isolate new myxobacterial groups are a permanent challenge.

Several studies dealt with the evaluation of myxobacteria exclusively detected with cultivation‐independent methods, for example, in lake mud (Li et al. 2012) or with cultivable myxobacteria in comparison to those detectable with cultivation‐independent methods (Wu et al. 2005; Jiang et al. 2007, 2010). In comparison, in our study, we assess the diversity of myxobacteria from two totally different samples (Kiritimati Island and German compost) instead of one sample as with Wu et al. 2005 and Jiang et al. 2007; with clone libraries of 16S rRNA genes as well as by cultivation. Therefore, a semiselective primer pair from the literature (Wu et al. 2005), with forward primer specific for Sorangiineae/Nannocystineae and Cystobacterineae, respectively, and eubacterial reverse primer was used for PCR. The sequences were compared to the 16S rRNA genes of the received cultures and to sequences of a public database (GenBank). All sequences received in this study were integrated into a phylogenetic tree constructed of sequences from all myxobacterial‐type strains. Herewith, we provide new insight into the cosmopolite distribution of some myxobacterial genera as well as the existence and distribution of hitherto unidentified and therefore highly interesting myxobacteria with respect to their potential as producers of new secondary metabolites.

## Experimental Procedures

### Soil sample collection

The sample from Kiritimati was taken in March of 2011, 10 m inland from the edge of the hypersaline Lake 21 (01°96′N, 157°33′W; Schneider et al. 2013) on the Kiritimati atoll (Northern Line Islands, Republic of Kiribati, Central Pacific). No plant residues were visible in this sandy sample. Compost samples were collected in May 2012 from different parts of a free‐standing compost heap (Groß Biewende, Lower Saxony, Germany; 52°05′N, 10°37′E), combined and homogenized afterwards. Kiritimati and compost samples were dried at 30°C and stored at room temperature.

### Isolation techniques

Predatory and cellulolytic myxobacteria were isolated by the methods of Shimkets et al. (2006) and also with modified approaches. In addition to the standard techniques (cultivation from air‐dried sample material at 30°C in a dark breeding chamber on water agar with living *E. coli* and Stan 21 agar with filter paper; pH 7.2), exposure of moist sample material, dried sample at different temperatures (room temperature, 40°C), pH values (4.5, 6.5, 8.0), cultivation under sun exposure, with 5% CO_2_ at 30°C, with addition of 1% compost extract, or 2% NaCl to the agar were performed. Two anaerobic appendages per sample were conducted using water agar with autoclaved *E. coli* and Stan 21 agar with filter paper using the Anaerocult^®^ P‐system (Merck, Darmstadt, Germany). The anaerobic approaches were incubated at 30°C for 3 months. All in all, the two soil samples were applicated on 184 agar plates, including 51 multiple tests. The compost sample was applicated on 156 plates, and the small volume Kiritimati sample on 28 plates. The appearance of myxobacterial swarms or fruiting bodies were detected under a dissecting microscope every few days. Fruiting bodies or material from swarm edges were transferred to new water agar plates with *E. coli* and finally to CY or VY/2 agar, respectively (Reichenbach and Dworkin, 1992). Pure cultures were transferred from the agar plate to 20 mL CY/H‐medium [per liter: 1.0 g defatted soy flour, 1.0 g glucose, 4.0 g starch (Cerestar), 1.5 g yeast extract, 1.5 g casitone, 1.0 g CaCl_2_ • 2H_2_O, 0.5 g MgSO_4_ • 7H_2_O, 0.008 g iron EDTA, 11.8 g HEPES, pH 7.3]. Portions of 1.5 mL of well‐grown cultures were conserved at −80°C.

### DNA extraction from cultures

DNA of pure cultures was extracted with the Spin Plant Mini Kit (Invisorb, Birkenfeld, Germany). Therefore, 1.5 mL from a well‐grown liquid culture was centrifuged in an Eppendorf cup and the supernatant was removed. Afterwards, 100 *μ*L lyses buffer from the kit was added to the pellet. The whole mixture mashed up and was incubated for 5 min at 95°C. The remaining steps were performed according to the provider's instruction.

### PCR conditions for amplification of 16S rRNA genes from cultures

A first PCR reaction was started with eubacterial primers F27/R1525 in a volume of 50 *μ*L: 25 *μ*L JumpStart Taq ReadyMix (Sigma‐Aldrich, Munich, Germany), 0.4 *μ*Mol/L of each primer (final concentration), 2 *μ*L genomic DNA, 19 *μ*L PCR water. PCR amplifications were conducted in a Mastercycler Gradient (Eppendorf, Hamburg, Germany) using the following conditions: initial denaturation at 95°C (5 min); 35 cycles of denaturing at 94°C (30 sec); annealing at 52°C (30 sec); extension at 72°C (120 sec); and a final extension at 72°C (600 sec). PCR products were checked on an agarose gel (0.8%), purified using the NucleoSpin^®^ Gel and PCR Clean‐up‐Kit (Macherey‐Nagel, Düren, Germany) and eluted in 30 *μ*L elution buffer.

### Sequence analyses

For a first assignment, 16S rRNA genes of the cultures were sequenced using primer F27 and R518. For six cultivated representatives of the OTUs (C8, B1, C6, C4, B17, C17), full‐length 16S rRNA genes were sequenced using additional primer F357 (Muyzer et al. 1993), F945, R1078, and R1525 to assure that both nucleotide directions were covered. The sequences of these six cultures have been deposited at GenBank under accession numbers KP18974–KP18979. The specificity of the two primer combinations (FW2/FW5 and R1525) was checked by PCR using genomic DNA of 21 representatives derived from the phyla Actinobacteria, Cyanobacteria, Firmicutes, *α*‐, *β*‐, and *γ*‐Proteobacteria (Table S1), and additional 22 representatives of the order Myxococcales (Table S2). PCR applicability of extracted DNA from the representatives was initially checked with eubacterial primers F27/R1525 as described for the isolated strains. Afterwards, the PCR conditions for the semispecific myxobacterial primer sets (FW2/FW5 ‐ R1525) were tested. The annealing temperature was optimized using sequential gradient PCR reactions, starting with a temperature range between 50°C and 70°C. The PCR products were checked via agarose gel and the temperature range was narrowed down until an optimal annealing temperature for both primer sets could be defined, such as 61.1°C for combination FW2/R1525 and 65.5°C for FW5/R1525. These conditions were used for the amplification of myxobacterial 16S rRNA genes in the further process.

### DNA extraction from soil

Total DNA was extracted from soil using the UltraClean^®^ Soil DNA Kit (Mobio, Carlsbad, USA) following the manufacturer's instruction. Triplicates (each 1 g) of both samples were extracted in parallel and the quality of genomic DNA was checked by agarose gel (0.8%). High‐quality DNA was combined and used for PCR.

### PCR amplification of 16S rRNA genes from soil samples

Humid acids are common in soil and can inhibit PCR reactions. To test the PCR proficiency, the extracted genomic DNA was initially used in a PCR with universal bacterial primer F27/R1525 (Lane 1991; Stackebrandt et al. 1993) as described for the cultures. For the myxobacteria‐specific clone bank analyses, two semi specific primer pairs have been chosen from the literature, each with the universal reverse primer R1525 and a myxobacteria specific forward primer FW5 (Wu et al. 2005) for Sorangiineae/Nannocystineae, resulting in an amplicon of 996 bp and FW2 (Wu et al. 2005) for Cystobacterineae amplifying 1098 bp. The direct use of these semispecific primer set FW2/R1525 with genomic soil DNA (in triplicate) resulted in PCR products of the expected length. The same approach with primer set FW5/R1525 failed. Subsequent, the PCR product of the eubacterial approach (F27/R1525) was diluted 1:10 with PCR water and used as a template for a nested PCR (in triplicate) with both semispecific myxobacterial primer sets. The PCR conditions were as described above, but with 25 cycles and an annealing temperature of 61.1°C for the set FW2/R1525 and 65.5°C for FW5/R1525. PCR products were checked on agarose gel and purified using the NucleoSpin Gel and PCR clean up Kit (Macherey Nagel). In the case of primer set FW2/R1525, both the PCR products from the direct and the nested PCR were combined. For more description of the primer used in this study see Table S3. Amplified PCR products were purified as described above, ligated and transformed into competent *E. coli* cells JM109 using the pGEM^®^‐T Easy Vector System (Promega, Mannheim, Germany) with subsequent blue–white screening as recommended by the manufacturer. For each of the both samples, two libraries of 16S rRNA gene sequences were thus established: the Cystobacterineae enriched library (FW2/R1525) and the Sorangiineae/Nannocystineae enriched library (FW5/R1525). Material of white clones was picked with a sterile tooth pick and directly introduced into a PCR reaction with primer pair pUC/M13 (Promega). PCR conditions were as recommended by the manufacturer of the kit (Promega). PCR products were checked on an agarose gel and a total of 200 products of the expected length were chosen (50 per library) for sequencing using primer FW2 or FW5, respectively. Only sequences of high quality and length (>500 kb) were analyzed.

### Data analysis

The 16S rRNA gene sequences acquired via clone bank and from the isolates were checked for quality using the program SeqManII. The sequences from the cultures were assembled into consensus sequences. The single sequences from clones as well as the consensus sequences from cultures were compared with the NCBI database entries (FASTA; http://www.ebi.ac.uk/Tools/sss/fasta/). Closely related myxobacterial sequences, representing different species, were imported into the ARB database (Ludwig et al. 2004; version 14.02.2005 database; http://www.arb-home.de) and aligned together with the sequences acquired in this study. A distance matrix tree was constructed with 16S rRNA sequences of the 51 myxobacterial‐type strains using the Neighbor‐Joining method (Saitou and Nei 1987) and Jukes Cantor correction (Jukes and Cantor 1969). The topology of the phylogenetic tree was built by bootstrap analysis of 1000 operations. As recommended in ARB, no filter was used in bootstrapping. Sequences sharing more than 99% similarity, calculated with the similarity matrix tool in ARB, were grouped into operational taxonomic units (OTUs). For phylogenetic analyses, the 16S rRNA gene of a cultivated representative of each OTU, if there was one, was fully sequenced. Only the type and representative myxobacterial strains as well as clones of uncultured bacteria with highest similarity to the novel isolates were shown in the phylogenetic tree. Table S4 shows the type strains and their corresponding 16S rRNA gene accession numbers used for the construction of the phylogenetic tree.

### Nucleotide sequence accession numbers

Sequences of clones were deposited in GenBank under accession numbers KJ475674–KJ475752, those of representative cultures under numbers KP18974–KP18979.

## Results

### Cultivation of myxobacteria from Kiritimati sand and German compost

Using enhanced standard cultivation methods on 184 agar plates, more than 100 predatory myxobacterial strains could be isolated. Based on morphological characteristics, we tried to reduce the number of replicates. Finally, a total of 42 cultures, 17 from Kiritimati and 25 from the compost sample were included in this study (Table 2 and Table S5). On basis of 16S rRNA gene sequences, 41 cultures were grouped into three operational taxonomic units (OTUs; ≥99% similarity); *Myxococcus* spp. (Cb1), *Corallococcus* spp. (Cb2), and *Archangium gephyra* clade (Cb3). *Polyangium fumosum* is a single sequence which belongs to the Sorangiineae. The origins of these cultures are listed in Table 1. Based on 16S rRNA gene analyses the 17 strains from Kiritimati belong to Myxococcaceae (*Myxococcus* spp.) and Cystobacteraceae (99.1% sequence homology to the type strain^(T)^ of *Archangium gephyra*). The compost strains classed into Myxococcaceae (*Myxococcus* spp.; *Corallococcus* spp.) and Polyangiaceae (*Polyangium fumosum* ^T^; 99.2%). From representatives of OTUs Cb1 (B1, C6, C8), Cb2 (C4), Cb3 (B17) and from the Sorangiineae culture C17, the 16S rRNA genes were almost completely sequenced and deposited at GenBank (accession numbers see Table S4).

**Table 1:** Origin of clones achieved in this study with Cy (Cystobacterineae) specific forward primer FW2 and Na/So (Nannocystineae/Sorangiineae) specific forward primer FW5; B, Kiritimati; C, German compost

Primer/origin of clones	B	C	Total
FW2‐R1525 (Cy)	31	29	60
FW5‐R1525 (Na/So)			
Nannocystineae	4	0	4
Sorangiineae	11	4	15
Total	46	33	79

**Figure 1:**
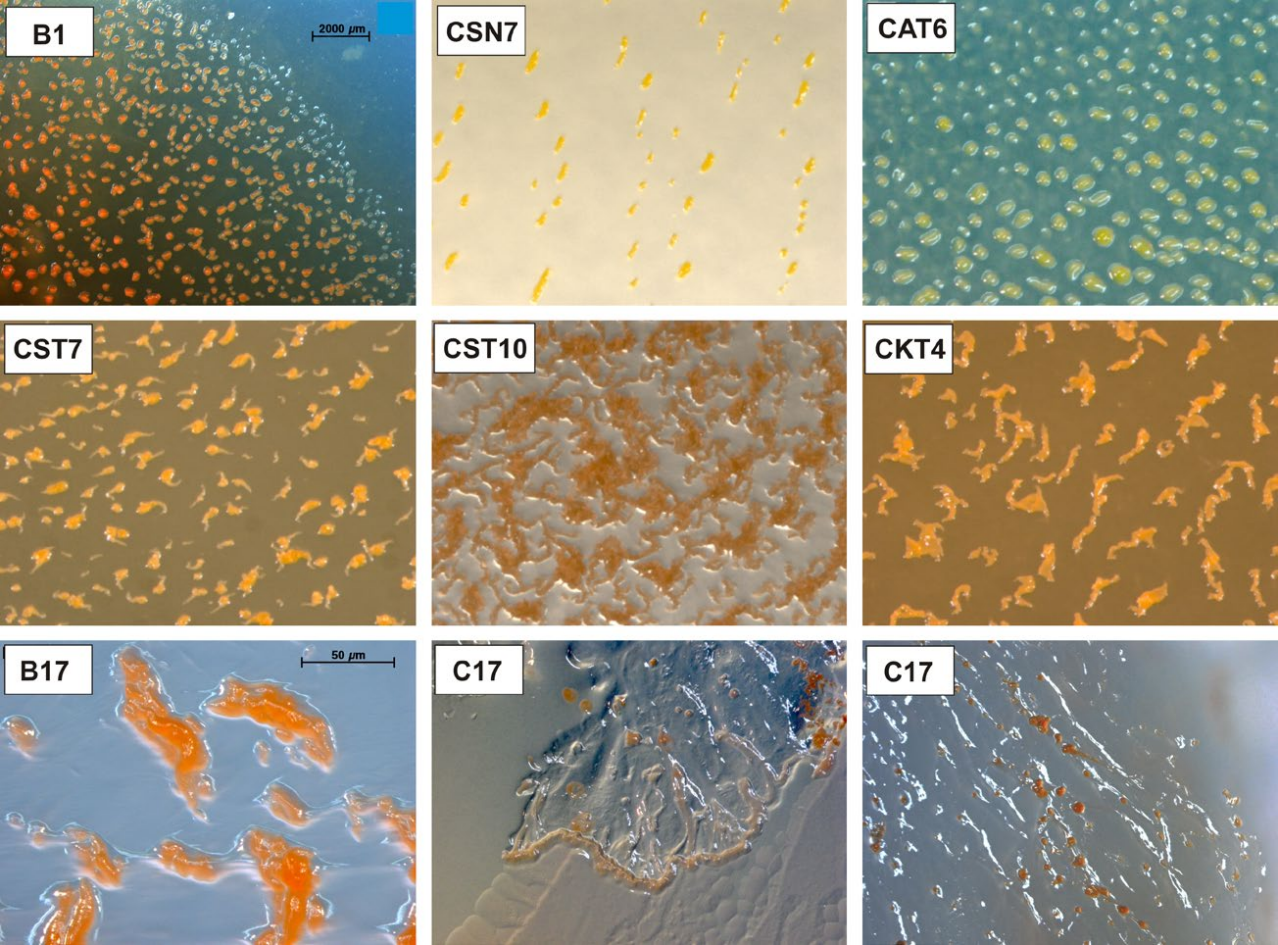
Representatives of the *Myxococcus* clade (operational taxonomic units: Cb1; upper row), *Corallococcus* clade (OTU Cb2; middle row), *Archangium* clade (B17; OTU Cb3; bottom row), and *Polyangium* clade (C17; single sequence within Sorangiineae; bottom row. Photo in the middle: swarm on water agar degrading *E. coli*). All other cultures are on VY/2‐agar. B, from Kiritimati; C, from German compost sample.

Microphotographs of representatives of each clade are shown in Figure 1. The nine cultures from the *Corallococcus* clade (all from compost) were achieved with standard methods as well as with incubation at 20°C or light exposure and addition of compost extract to the agar. Culture B17 was isolated from water agar with *E. coli*. No cellulolytic myxobacteria like *Sorangium cellulosum* or *Byssovorax cruenta* could be isolated.

### Establishment of myxobacterial 16S rRNA gene libraries from soil samples

A total of 121 sequences were achieved by clone bank analyses. Seventy‐nine sequences group to Myxococcales. The other 42 sequences were excluded from our study. The sequence lengths range from 519 to 809 bp. Fourty‐six clones emerged from the Kiritimati sample (B) and 33 from German compost (C; Table 1). A total of 60 clones grouped into the suborder Cystobacterineae and the remaining 19 sequences into the Nannocystineae/Sorangiineae.

The forward primer specificity within the Myxococcales was perfect: all clones which grouped into the Cystobacterineae originate from PCR products amplified with forward primer FW2, (specific for Cystobacterineae) and all clones which grouped into the Nannocystineae/Sorangiineae were from PCR products amplified with forward primer FW5, (specific for Nannocystineae/Sorangiineae). The clones distribute among 12 operational taxonomic units (OTUs) based on 99% sequence similarity as shown in Table 2. Also shown in Table 2 are the culture affiliations to the OTUs.

**Table 2:** Number and source of clones and cultures of operational taxonomic units (OTUs). Cystobacterineae (Cb), Nannocystineae (Na) and Sorangiineae (So). “Single” sequences show less than 99% similarity to the remaining sequences. B, Kiritimati; C, German compost

OTU	Clones			Cultures		
	B	C	Total	B	C	Total
Cb1 *Myxococcus*	1	1	2	16	15	31
Cb2 *Corallococcus*	6	0	6	0	9	9
Cb3 *Archangium gephyra*	6	1	7	1	0	1
Cb4	3	15	18	0	0	0
Cb5	2	0	2	0	0	0
Cb6	0	3	3	0	0	0
Cb7	2	0	2	0	0	0
Cb single	11	9	20	0	0	0
Na1	2	0	2	0	0	0
Na2	2	0	2	0	0	0
So1	2	0	2	0	0	0
So2	3	0	3	0	0	0
So3	0	2	2	0	0	0
So single	6	2	8	0	1	1
Total	46	33	79	17	25	42

**Figure 2:**
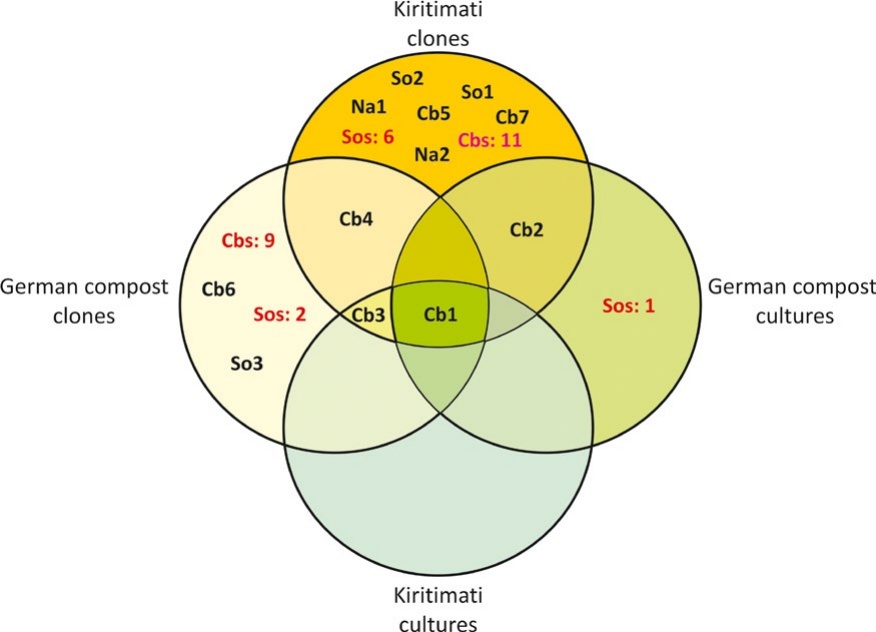
Venn diagram representing OTUs composed of clones and cultures from Kiritimati (B) and German compost (C). Operational taxonomic units (OTU) belong to Cb, Cystobacterineae; So, Sorangiineae; Na, Nannocystineae. Number of single sequences belonging to Cbs: X, Cystobacterineae; Sos: X, Sorangiineae.

A total of 51 clone sequences grouped into the 12 OTUs, which contain at least two sequences with ≥99% similarity. With three exceptions (Cb1, Cb3, Cb4), the OTUs contain exclusively clones from one sampling site. Twenty‐eight sequences showed less than 99% similarity and were designated as “single”. The 29 nonsingle clones from Kiritimati distributed among 10 different OTUs whereby the 22 nonsingle clones from compost merely grouped into five OTUs (Fig. 2).

Non‐Myxococcales sequences were excluded from our analyses (data not shown). Single sequences or sequences of clones from OTUs which did not include type strain sequences were blasted in GenBank to figure out the next cultivated relative. In all cases, the next cultivated relative belongs to the Myxococcales. So, we are sure that all our clone sequences included in this study are of myxobacterial origin.

### Comparison of cultures and clones received in this study

**Figure 3:**
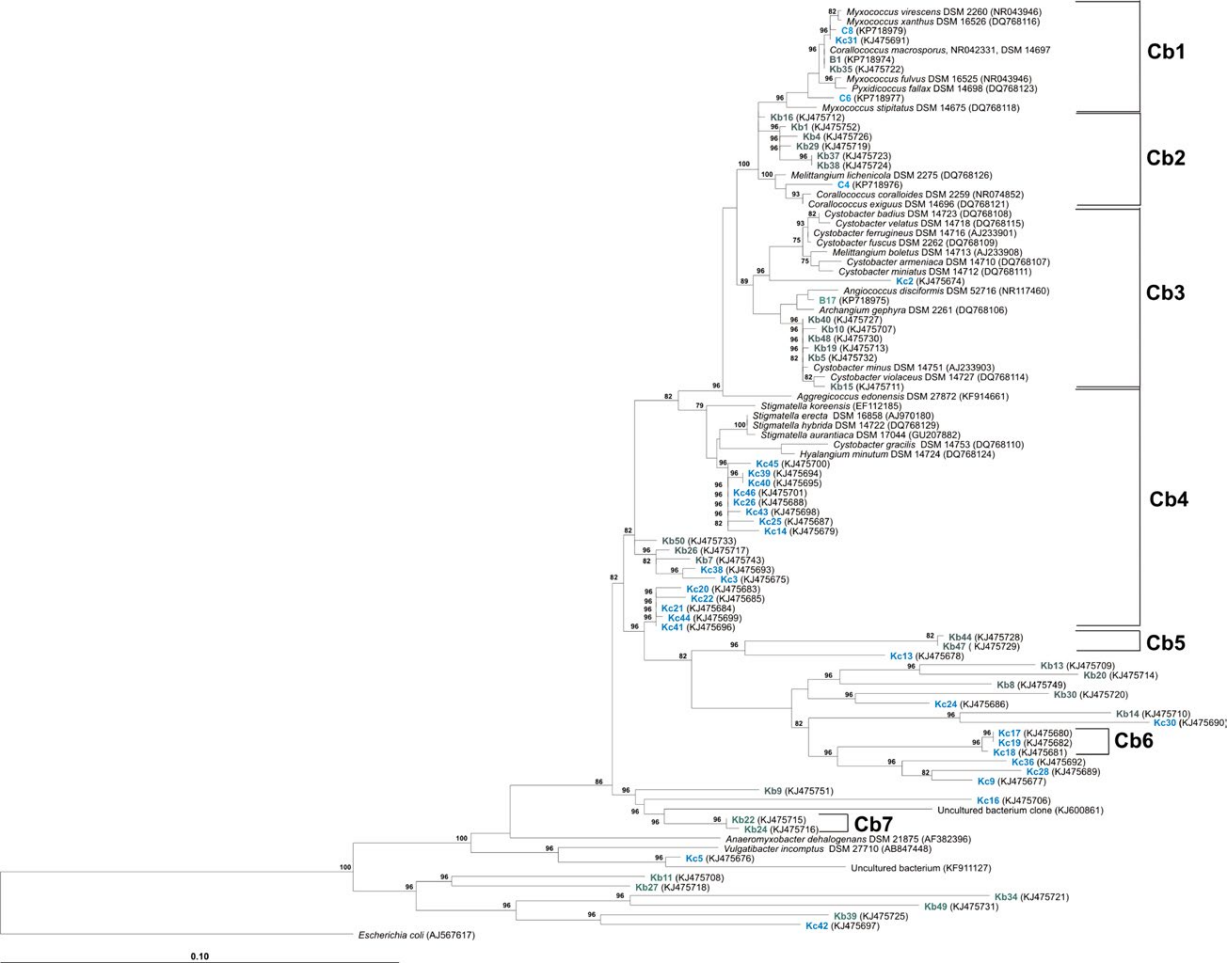
Distance matrix tree based on 16S rRNA‐gene sequences of Cystobacterineae (type strains), clones from this study (Kb, Kiritimati; Kc, German compost), representative cultures from Kiritimati (B) and German compost (C) and some related clones from other studies. Operational taxonomic units (OTUs) Cb1 ‐ Cb7 are indicated. Numbers at the branch points indicate bootstrap support based on 1000 resamplings. Bootstrap values less than 70% are not shown. Bar, 0.10 substitutions per nucleotide position. The GenBank accession numbers of sequences used in the tree are shown in parentheses.

From 12 OTUs, three Cystobacterineae clades were represented by clones as well as by cultures from this study (Cb1‐3; Table 2), whereby exclusively the *Myxococcus* clade Cb1 was represented by clones and cultures from Kiritimati and from German compost. Cb4 was not represented by cultures from this study, but by several type strain sequences of Cystobacteraceae. The sequences of OTUs Cb5–Cb7 as well as all single‐clone sequences showed no remarkable relationship with cultivated strains at all (Figs. 2 and 3).

**Figure 4:**
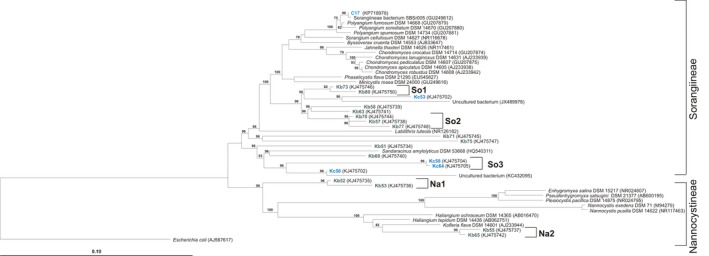
Distance matrix tree based on 16S rRNA gene sequences of Sorangiineae/Nannocystineae (So/No) (type strains), clones from this study (Kb, Kiritimati; Kc, German compost), representative culture from German compost (C) and some related clones from other studies. Operational taxonomic units (OTUs) (So/Na) are indicated. Numbers at the branch points indicate bootstrap support based on 1000 resamplings. Bootstrap values less than 70% are not shown. Bar, 0.10 substitutions per nucleotide position. The GenBank accession numbers of sequences used in the tree are shown in parentheses.

The Nannocystineae was exclusively represented by clones from Kiritimati, grouped into two OTUs (*n*: 4; Table 2). The Sorangiineae suborder was mainly represented by clones from Kiritimati (*n*: 11) in comparison to clones from compost (*n*: 4). The only Sorangiineae culture from this study (C17) was related to *Polyangium fumosum*, isolated from compost and exclusively detected by cultivation (Figs. 2 and 4). However, none of the clone sequences could be associated with sequences of Sorangiineae/Nannocystineae type strains.

## Discussion and Conclusion

Myxobacteria belong to the most interesting groups of natural product producers. New groups of myxobacteria frequently lead to new secondary metabolites, which often show bioactivity. Because of the special urgent need for new antibiotics, in particular undescribed groups of myxobacteria are in the focus of our isolation activities. To consider whether standard isolation procedures, established in our lab for more than 20 years, are suitable for the actually present myxobacterial diversity in two completely different environmental samples, we compared 16S rRNA genes of cultivable myxobacteria to those detected with cultivation‐independent clone bank analyses. Therefore, a sandy sample from Kiritimati Island as well as a sample from German compost was analyzed.

From the 42 cultures of this study 40 belong to the Myxococcaceae (*Myxococcus*,* Corallococcus*), one to the Cystobacteraceae (*Archangium*) and one to the Polyangiaceae (Polyangium). But, especially myxobacteria of the genera *Myxococcus* and *Corallococcus* are, in some cases, difficult to differentiate on basis of their 16S rRNA genes (Stackebrandt et al. 2007). Therefore, the 31 cultures of *Myxococcus* OTU Cb1, isolated from both sites, as well as the nine cultures of *Corallococcus* OTU Cb2, isolated exclusively from compost, probably represent more than two species. Singh already described in 1947 *Myxococcus fulvus* to be dominant in active compost and *M. virescens* and *Corallococcus* (formerly *Chondrococcus*) *exiguus* as at least present in this habitat. Twenty‐four of 25 cultures isolated from German compost grouped into the *Myxococcus*/*Corallococcus* OTUs Cb1 and Cb2, respectively. Sixteen of 17 cultures from the Kiritimati sample are located in Cb1. The remaining two cultures are *Archangium gephyra* from Kiritimati and *Polyangium fumosum* from compost. Despite the large discrepancy in the number of cultivation approaches (28 agar plates with sand from Kiritimati and 156 agar plates with German compost), the diversity of cultivable myxobacteria detected with enhanced standard methods is comparable for both sampling sites (see Experimental Procedures).

Examined in more detail, the Cystobacterineae are moderately represented by both approaches: Cb1, containing sequences of all four *Myxococcus* type strains, as well as *Pyxidicoccus fallax* and *C. macrosporus*, is well represented by clones and cultures from both sampling sites. The standard cultivation methods are suitable for members of this OTU. Cb2 was represented by clones from Kiritimati and cultures exclusively isolated from compost. Within this OTU, clones and cultures represent at least two different strains. Cb3 is represented by clones and one culture from Kiritimati and one clone from compost. For Cb2 and Cb3, the isolation success can be considered as moderate. Cb4 was represented by type strain sequences as well as by clones from both sites. Cb5–Cb7 as well as the 20 single sequences of the Cystobacterineae show no relationship with cultures at all. For all of these myxobacteria, our enhanced standard cultivation techniques are insufficient.

The suborders Nannocystineae/Sorangiineae are poorly represented in this study. Fifteen of 19 clones in these suborders are from Kiritimati. The only culture (C17) was isolated from compost and belongs to the Sorangiineae. Exclusively bacteriolytic, but no cellulolytic myxobacteria like *Sorangium cellulosum* or *Byssovorax cruenta* could be isolated or detected. For the sandy sample of Kiritimati, where no plant material was visible, this result is not surprising, but in compost, we would have expected cellulolytic myxobacteria.

All in all, the diversity of myxobacteria detected with the molecular method is much higher (12 OTUs and 28 single sequences) than with extended standard cultivation methods (3 OTUs and one single sequence). This result is in accordance with other studies in which cultivable strains were compared to those detected with cultivation‐independent methods (Wu et al. 2005; Jiang et al. 2010). Jiang et al. (2007), for example, surveyed the diversity of myxobacteria present in campus garden soil by both cultivation‐based and cultivation‐independent methods. Their cultivated, fruiting‐body producing strains belong to known taxa, which comprised only in a small part of the sequences recovered directly from soil in the cultivation‐independent approach. As in our study, Jiang et al. (2007) concluded that the myxobacterial group is much more diverse than previously thought. In addition, the colleagues suggest that myxobacteria exist in two forms: the fruiting and the nonfruiting types. The authors consider that most of the uncultured myxobacteria may represent taxa which rarely form fruiting bodies. But, the authors also assume that from the described myxobacteria, only fruiting bodies were isolated from the environmental samples placed on agar. In principle, we isolate fruiting bodies as well as swarm edges from strains without fruiting body production. To the best of our knowledge, no immotile myxobacterial species are described. We assume that myxobacteria detected with molecular, but not with cultivation methods were present in the sample as vegetative cell form and not in a dry‐resistant myxospore state. We cannot make any statement if these myxobacteria are able to convert into myxospores/fruiting bodies or not, or if the nutritional circumstances of the environmental sample before collection were so suitable that there was no need for the myxobacteria to convert to a resting stage. Normally, we dry environmental samples at room temperature or 30°C to minimize fungal or nonmyxobacterial contaminations. The compost sample was also applied without drying, but the cultivation success was not higher. Jiang also suggested that the growth requirements in the laboratory are not sufficient for the hitherto uncultivated species. We think that this is the main reason for the failure in cultivating new myxobacterial species within this study.

Myxobacteria grow, in comparison to other soil inhabiting bacteria like Actinobacteria, slow, and according to Wu et al. (2005), there are less than 1% myxobacteria in number among soil bacteria. Although we added fungicides like soraphen and cycloheximide to the isolation agar, fungal contaminants as well as other soil bacteria often overgrow the sample before myxobacteria are visible. Myxobacteria consume macromolecules by enzymatic degradation. They practice a “social” lifestyle, where thousands of cells share their enzymes for digestive function. However, single or few cells are hardly to isolate on water agar/*E. coli* or Stan21/filter because they are probably not able to exclude enough enzymes to make nutrients accessible for growth and cell division activities. So, even if few cells of still undiscovered myxobacteria exist in a soil sample, although detectable with molecular methods, they will not be able to proliferate in visible amounts, the prerequisite for isolation.

The myxobacterial diversity detected by clone bank analyses is about twice as high in the Kiritimati sample (ten OTUs and 17 single sequences) as in the compost sample (five OTUs and 11 single sequences). This result is surprising because the Kiritimati sample was sandy without plant residues and compost is known as a hot spot for bacterial communities. On the other hand, the presence of members of Nannocystineae in Kiritimati, but not in compost is not remarkable. The Kiritimati sample was collected near the hypersaline Lake 21 and most genera of the Nannocystineae suborder were described as halotolerant or halophilic like *Plesiocystis* (Iizuka et al. 2003a), *Enhygromyxa* (Iizuka et al. 2003b), *Pseudenhygromyxa* (Iizuka et al. 2013), and *Haliangium* (Fudou et al. 2002).

The results of this work indicate that some genera like *Myxococcus* and *Corallococcus* are well covered with our extended standard cultivation methods. However, other groups are exclusively composed of clones from just one or both sampling sites, and show no relationship with any known, cultivated myxobacterium. The cultures, with one exception, were also detected with clone bank analyses on a high level of similarity, demonstrating that the molecular approach works well for the cultivable species. Although only few myxobacterial genera were obtained by culturing methods, molecular surveys revealed many myxobacteria related 16S rRNA gene sequences in sand from Kiritimati and German compost, which were phylogenetically similar in some clades, but mostly separated from known cultivated myxobacteria. Maybe methods like cultivation in diffusion chambers or isolation chips (ichip) could be suitable for myxobacteria, too. Kaeberlein et al. (2002) were successful in cultivating previously uncultivated microorganisms in a diffusion chamber simulating natural environment. Their isolated marine organisms were not able to grow on artificial media alone, but formed colonies in the presence of other microorganisms. Nichols et al. (2010) enhanced this method by designing an ichip composed of several hundred miniature diffusion chambers, each inoculated with a single‐environmental cell. With this method also a new antibiotic, teixobactin, could be discovered in a screen of uncultured bacteria by Ling et al. (2015).

Our study takes on the well‐known problem of the discrepancy of microorganisms present in a sample (whether it is soil or water or something else) and those gettable by standardized isolation methods. Due to the strong need of new antibiotics, we will focus on the development of new isolation methods for hitherto uncultivated myxobacteria, a promising source for new bioactive compounds.

## Conflict of Interest

None declared.

## Supporting information


**Table S1.** List of representatives derived from the phyla Actinobacteria, Cyanobacteria, Firmicutes, *α*‐, *β*‐, and *γ*‐Proteobacteria to check specificity of the two primer combinations FW2/FW5 and R1525.
**Table S2.** DNA of the listed representatives of Myxococcales was used for optimization of annealing temperature of different primer combinations.
**Table S3.** Primers used in this study.
**Table S4**. All type strains of valid described myxobacterial species with DSM‐ and Accession number used for the construction of the phylogenetic core tree, representative cultures of each OTU and additional sequences with high similarity to sequences of this study.
**Table S5**. Cultures established in this study and affiliation to OTUs based on 99% sequence similarity.
