# Supplementary material for: Comparison of myxobacterial diversity and evaluation of isolation success in two niches: Kiritimati Island and German compost
Source: Microbiologyopen. 2015 Dec 15;5(2):268–78. doi: 10.1002/mbo3.325 (PMC4831471; doi:10.1002/mbo3.325)
Supplement: Supplementary file 1 — Table S1. List of representatives derived from the phyla Actinobacteria, Cyanobacteria, Firmicutes, α‐, β‐, and γ‐Proteobacteria to check specificity of the two primer combinations FW2/FW5 and R1525. Table S2. DNA of the listed representatives of Myxococcales was used for optimization of annealing temperature of different primer combinations. Table S3. Primers used in this study. Table S4. All type strains of valid described myxobacterial species with DSM‐ and Accession number used for the construction of the phylogenetic core tree, representative cultures of each OTU and additional sequences with high similarity to sequences of this study. Table S5. Cultures established in this study and affiliation to OTUs based on 99% sequence similarity. [file MBO3-5-268-s001.docx]

**Comparison of Myxobacterial Diversity and Evaluation of Isolation Success in two niches: Kiritimati Island and German Compost**

**Kathrin Irene Mohr,^1^ Marc Stechling,^2,3^ Joachim Wink,^2^ Elke Wilharm^3^, and Marc Stadler ^1,^***

^1^Microbial Drugs, Inhoffenstrasse 7, D-38124 Braunschweig, Germany, ^2^Microbial Strain Collection, Helmholtz Centre for Infection Research, Inhoffenstrasse 7, D-38124 Braunschweig, Germany, ^3^Department of Supply Engineering, Ostfalia, Salzdahlumer Straße 46/48  D-38302 Wolfenbüttel, Germany.

*For correspondence. E-mail [marc.stadler@helmholtz-hzi.de](mailto:marc.stadler@helmholtz-hzi.de); Tel. +49 531 6181 4240; Fax +49 6181 9499

| Table S1. List of representatives derived from the phyla Actinobacteria, Cyanobacteria, Firmicutes, α-, β-, and γ-Proteobacteria to check specificity of the two primer combinations FW2/FW5 and R1525.Phylum | Class | Order/Suborder | Genus |
| --- | --- | --- | --- |
| Actinobacteria | Actinomycetales | Corynebacterineae | *Mycobacterium lacticola* |
|  |  |  | *Nocardia alboflava* |
|  |  |  | *Rhodococcus opacus* |
|  |  |  | *Corynebacterium mediolanum* |
|  |  |  | *Corynebacterium fascians* |
|  |  | Micrococcineae | *Micrococcus luteus* |
|  |  |  | *Arthrobacter rubellus* |
|  |  |  | *Corynebacterium ammoniagenes* |
| Cyanobacteria | Cyanobacteria | Oscillatoriales | *Coleofasciculus chthonoplastes* |
|  |  | Nostocales | *Nostoc punctiforme* |
|  |  |  | *Rivularia sp.* |
|  |  | Chroococcales | *Aphanocapsa muscicola* |
| Firmicutes | Bacilli | Bacillales | *Bacillus thuringiensis* |
|  |  |  | *Staphylococcus aureus* |
| Proteobacteria | Alphaproteobacteria | Rhodobacterales | *Roseobacter sp.* |
|  | Betaproteobacteria | [Neisseriales](http://www.ncbi.nlm.nih.gov/Taxonomy/Browser/wwwtax.cgi?mode=Undef&id=206351&lvl=3&lin=f&keep=1&srchmode=1&unlock) | *Chromobacterium violaceum* |
|  | Gammaproteobacteria | Enterobacteriales | *Escherichia coli* |
|  |  |  | *Klebsiella* sp. |
|  |  |  | *Salmonella typhi* |
|  |  |  | *Serratia marcescens* |
|  |  | Pseudomonadales | *Pseudomonas aeruginosa* |

| Table S2. DNA of the listed representatives of Myxococcales was used for optimization of annealing temperature of different primer combinations.Cystobacterineae | Sorangiineae |
| --- | --- |
| *Archangium* sp. (Ar4759) | *Byssovorax cruenta* (Byc2; DSM 14553^T^) |
| *Corallococcus coralloides* (Ccc97) | *Chondromyces crocatus* (Cmc2; DSM 14606) |
| *Cystobacter velatus* (Cbv34; DSM 14718^T^) | „*Haploangium rugiseptum*“ (Har1; MINS) |
| *Hyalangium minutum* (NOCb2; DSM 14724^T^) | *Jahnella thaxteri* (Plt330; MINS) |
| *Melittangium lichenicola* (Mel13; DSM 52905) | *Polyangium fumosum* (PI 31007) |
| *Myxococcus xanthus* (Mxx88) | *Sandaracinus amylolyticus* (NOSo 4; DSM 53668 ^T^) |
| NOCb22*; MINS | 706KM**; (MINS) |
| NOCb26*; MINS | *Sorangium cellulosum* (Soce1267; MINS) |
| *Pyxidicoccus fallax* (Ang983) | Nannocystineae |
| *Stigmatella aurantiaca* (Sga15) | *Haliangium ochraceum* (DSM 14365 T) |
|  | *Kofleria* sp. (Plvt8; DSM 53745) |
|  | *Nannocystis exedens* (Nae1067; MINS) |
|  | *Nannocystis pusilla* (Nap3; DSM 53155) |

* new organism of Cystobacteraceae; ** new organism of Sorangiineae; MINS: strain of the internal HZI strain collection (http://www.helmholtz-hzi.de/en/research/research_topics/anti_infectives/mikrobielle_stammsammlung/our_research/)

Table S3. Primers used in this study

| primer | specificity | position^a^ | primer sequences (5‘-3‘) | references |
| --- | --- | --- | --- | --- |
| F27 | UB | 8-27 | AGA GTT TGA TCC TGG CTC AG | Lane *et al*. 1991 |
| R518 | UB | 518-537 | CGT ATT ACC GCG GCT GCT GG | Lane *et al*. 1991 |
| F357 | UB | 338-357 | ACT CCT ACG GGA GGC AGC AG | Muyzer *et al*. 1993 |
| F945 | UB | 927-945 | GGG CCC GCA CAA GCG GTG G | Lane *et al*. 1991 |
| R1525 | UB | 1525-1542 | AAG GAG GTG ATC CAG CCG CA | Stackebrandt *et al*. 1993 |
| FW2 | CS | 427–444 | GTA AAG CAC TTT CGA CCG | Wu *et al*. 2005 |
| FW5 | SN | 529–550 | GTA AGA CAG AGG GTG CAA ACG T | Wu *et al*. 2005 |
| pUC M13F | pGem-T |  | CGC CAG GGT TTT CCC AGT CAC GAC | Promega |
| pUC M13R | pGem-T |  | TCA CAC AGG AAA CAG CTA TGA C | Promega |

^a^Correspond to the sequence number of 16S rRNA in *Escherichia coli* (Brosius *et al*., 1978). UB, universal bacteria; CS, Cystobacterineae-specific; SN, Sorangiineae/Nannocystineae-specific; f: forward; r: reverse.

Table S4. All type-strains of valid described myxobacterial species with DSM- and Accession number used for the construction of the phylogenetic core tree, representative cultures of each OTU and additional sequences with high similarity to sequences of this study

| **Genus** | **Species** | **DSM** | **Acc.-no.** | |  |
| --- | --- | --- | --- | --- | --- |
| *Myxococcus* | *fulvus* | 16525 | NR043946 | |  |
|  | *stipitatus* | 14675 | DQ768118 | |  |
|  | *virescens* | 2260 | NR043946 | |  |
|  | *xanthus* | 16526 | DQ768116 | |  |
| *Kofleria* | *flava* | 14601 | AJ233944 | |  |
| *Corallococcus* | *coralloides* | 2259 | NR074852 | |  |
|  | *exiguus* | 14696 | DQ768121 | |  |
|  | *macrosporus* | 14697 | NR042331 | |  |
| *Archangium* | *gephyra* | 2261 | DQ768106 | |  |
| *Nannocystis* | *exedens* | 71 | M94279 | |  |
|  | *pusilla* | 14622 | NR117463 | |  |
| *Haliangium* | *ochraceum* | 14365 | AB016470 | |  |
|  | *tepidum* | 14436 | AB062751 | |  |
| *Melittangium* | *boletus* | 14713 | AJ233908 | |  |
|  | *lichenicola* | 2275 | DQ768126 | |  |
| *Stigmatella* | *aurantiaca* | 17044 | GU207882 | |  |
|  | *erecta* | 16858 | AJ970180 | |  |
|  | *hybrida* | 14722 | DQ768129 | |  |
|  | *armeniaca* | 14710 | DQ768107 | |  |
| *Cystobacter* | *badius* | 14723 | DQ768108 | |  |
|  | *ferrugineus* | 14716 | AJ233901 | |  |
|  | *fuscus* | 2262 | DQ768109 | |  |
|  | *gracilis* | 14753 | DQ768110 | |  |
|  | *miniatus* | 14712 | DQ768111 | |  |
|  | *minus* | 14751 | AJ233903 | |  |
|  | *velatus* | 14718 | DQ768115 | |  |
|  | *violaceus* | 14727 | DQ768114 | |  |
| *Pyxidicoccus* | *fallax* | 14698 | DQ768123 | |  |
| *Polyangium* | *sorediatum* | 14670 | GU207880 | |  |
|  | *fumosum* | 14668 | GU207879 | |  |
|  | *spumosum* | 14734 | GU207881 | |  |
| *Sorangium* | *cellulosum* | 14627 | NR116678 | |  |
| *Hyalangium* | *minutum* | 14724 | DQ768124 | |  |
| *Jahnella* | *thaxteri* | 14626 | NR117461 | |  |
| *Chondromyces* | *apiculatus* | 14605 | AJ233938 | |  |
|  | *crocatus* | 14714 | GU207874 | |  |
|  | *lanuginosus* | 14631 | AJ233939 | |  |
|  | *pediculatus* | 14607 | GU207875 | |  |
|  | *robustus* | 14608 | AJ233942 | |  |
| *Angiococcus* | *disciformis* | 52716 | NR117460 | |  |
| *Byssovorax* | *cruenta* | 14553 | AJ833647 | |  |
| *Sandaracinus* | *amylolyticus* | 53668 | HQ540311 | |  |
| *Plesiocystis* | *pacifica* | 14875 | NR024795 | |  |
| *Anaeromyxobacter* | *dehalogenans* | 21875 | AF382396 | |  |
| *Enhygromyxa* | *salina* | 15217 | NR024807 | |  |
| *Phaselicystis* | *flava* | 21295 | EU545827 | |  |
| *Pseudenhygromyxa* | *salsuginis* | 21377 | AB600195 | |  |
| *Aggregicoccus* | *edonensis* | 27872 | KF914661 | |  |
| *Vulgatibacter* | *incomptus* | 27710 | AB847448 | |  |
| *Labilithrix* | *luteola* | 27648 | NR126182 | |  |
| *Minicystis* | *rosea* | 24000 | GU249616 | |  |
| cultures emerged  from this work |  | *bp* | Acc.-no. | |  |
| *Myxococcus* | sp. (C8) | 1438 | KP718979 | |  |
| *Myxococcus* | sp. (B1) | 1430 | KP718974 | |  |
| *Myxococcaceae* | bacterium (C6) | 1436 | KP718977 | |  |
| *Corallococcus* | *coralloides* (C4) | 1414 | KP718976 | |  |
| *Archangium* | *gephyra* (B17) | 1418 | KP718975 | |  |
| *Polyangium* | *fumosum* (C17) | 1309 | KP718978 | |  |
| **further sequences** |  |  | |  | |
| „*Stigmatella koreensis*“ |  |  | | EF112185 | |
| uncultured bacterial clone 58A | | | | KJ600861 | |
| uncultured bacterial clone from compost | |  | | KF911127 | |
| culture SBSr005 |  |  | | GU249612 | |
| uncultured bacterial clone B065 | |  | | JX489978 | |
| uncultured bacterial clone SEAA1CG031 | |  | | KC432095 | |

Table S5. Cultures established in this study and affiliation to OTUs based on 99 % sequence similarity. B: Kiritimati; C: German compost. Cultures with fully sequenced 16S rRNA gene are bold. So: Sorangiineae

| **culture** | **OTU** | **culture** | **OTU** |
| --- | --- | --- | --- |
| **B1** | **CB1** | C9 | **Cb1** |
| B2 |  | C11 |  |
| B3 |  | C12 |  |
| B4 |  | C13 |  |
| B5 |  | C14 |  |
| B6 |  | C16 |  |
| B7 |  | CAT6 |  |
| B8 |  | CST9 |  |
| B9 |  | CSN7 |  |
| B10 |  | CSN10 |  |
| B11 |  | C1 | **CB2** |
| B12 |  | **C4** |  |
| B13 |  | C10 |  |
| B14 |  | C15 |  |
| B15 |  | CKT3 |  |
| B16 |  | CKT4 |  |
| C3 |  | CST3 |  |
| C5 |  | CST7 |  |
| **C6** |  | CST10 |  |
| C7 |  | **B17** | **Cb3** |
| **C8** |  | **C17** | So single |
|  |  |  |  |
